# Impact of an antimicrobial stewardship programme on antimicrobial utilization and the prevalence of MDR *Pseudomonas aeruginosa* in an acute care hospital in Qatar

**DOI:** 10.1093/jacamr/dlaa050

**Published:** 2020-08-07

**Authors:** Mazen A Sid Ahmed, Hamad Abdel Hadi, Sulieman Abu Jarir, Abdul Latif Al Khal, Muna A Al-Maslamani, Jana Jass, Emad Bashir Ibrahim, Hisham Ziglam

**Affiliations:** 1 Microbiology Division, Department of Laboratory Medicine and Pathology, Hamad Medical Corporation, Doha, Qatar; 2 The Life Science Centre-Biology, School of Science and Technology, Örebro University, Örebro, Sweden; 3 Departments of Infectious Diseases, Hamad Medical Corporation, Doha, Qatar; 4 Biomedical Research Center, Qatar University, Doha, Qatar

## Abstract

**Background:**

The excessive and inappropriate use of antibiotics is universal across all healthcare facilities. In Qatar there has been a substantial increase in antimicrobial consumption coupled with a significant rise in antimicrobial resistance (AMR). Antimicrobial stewardship programmes (ASPs) have become a standard intervention for effective optimization of antimicrobial prescribing.

**Methods:**

A before–after study was conducted in Hamad General Hospital (603 bed acute care hospital): 1 year before implementation of a comprehensive ASP compared with the following 2 years. The ASP included a hospital-wide pre-authorization requirement by infectious diseases physicians for all broad-spectrum antibiotics. Prevalence of MDR *Pseudomonas aeruginosa* was compared with antimicrobial consumption, calculated as DDD per 1000 patient-days (DDD/1000 PD). Susceptibility was determined using broth microdilution, as per CLSI guidelines. Antibiotic use was restricted through the ASP, as defined in the hospital’s antibiotic policy.

**Results:**

A total of 6501 clinical isolates of *P. aeruginosa* were collected prospectively over 3 years (2014–17). Susceptibility to certain antimicrobials improved after the ASP was implemented in August 2015. The prevalence of MDR *P. aeruginosa* showed a sustained decrease from 2014 (9%) to 2017 (5.46%) (*P = *0.019). There was a significant 23.9% reduction in studied antimicrobial consumption following ASP implementation (*P = *0.008). The yearly consumption of meropenem significantly decreased from 47.32 to 31.90 DDD/1000 PD (*P = *0.012), piperacillin/tazobactam from 45.35 to 32.67 DDD/1000 PD (*P < *0.001) and ciprofloxacin from 9.71 to 5.63 DDD/1000 PD (*P = *0.015) (from 2014 to 2017).

**Conclusions:**

The successful implementation of the ASP led to a significant reduction in rates of MDR *P. aeruginosa*, pointing towards the efficacy of the ASP in reducing AMR.

## Introduction

Healthcare-associated infections (HAIs) caused by Gram-negative bacteria (GNB) are of particular concern since more than 50% have been reported to be associated with MDR organisms. *Pseudomonas aeruginosa* in particular is associated with wide varieties of HAIs,^1^ while MDR *P. aeruginosa* has emerged as a serious nosocomial problem with significant morbidity and mortality, with limited available treatment options, leading to prolonged hospital stays with cost implications.^2–4^ It is widely known and accepted without argument that antibiotic overconsumption and misuse is the driving force behind the emergence of antimicrobial resistance (AMR). Between 2000 and 2015 there was a 65% global increase in antibiotic consumption, mainly in low- and middle-income countries.^5^^,^^6^ This rise has been mirrored by significant global rising trends in AMR, stemming mainly from increased antibiotic consumption.^5^ AMR is associated with significant morbidity and mortality as well as substantial economic consequences.^7^^,^^8^ For all these reasons, it has been advocated that all countries should have mechanisms to monitor local and national antibiotic consumption as well as develop time-dependent AMR monitoring and surveillance systems.^9^ Numerous professional, clinical and public health organizations have developed guidelines for antibiotic stewardship,^10–12^ which have been widely advocated to monitor and regulate the appropriate and judicious prescribing of antimicrobial agents.^13^ To date, there is growing evidence demonstrating the benefits of antimicrobial stewardship programmes (ASPs), including reductions in antimicrobial usage and cost. Pre-prescription approval and post-prescription review are two major types of ASP. Additional elements of ASP could include education, guidelines and clinical pathways. A major impetus for developing our programme was to improve the appropriateness of prescribing and preserve *P. aeruginosa* susceptibility, given the limited number of antibiotics with activity against this important pathogen.

According to internal monitoring records, Qatar has witnessed a decade of substantially rising antibiotic consumption, especially of carbapenems, coupled with a high prevalence of GNB isolated from patients at Hamad Medical Corporation (HMC) in Qatar, with *P. aeruginosa* being the second most prevalent. From 2009 to 2014 there was a noticeable increase in resistance to both colistin and meropenem (HMC’s antibiogram; internal publication, Laboratory Medicine and Pathology, Microbiology Division, HMC, 2009). In 2015, 8.1% of *P. aeruginosa* strains from the five major hospitals in Qatar were MDR.^14^ The objectives of this study were to describe how implementation of an institutional multimodal ASP affected the susceptibility of *P. aeruginosa*, the prevalence of MDR *P. aeruginosa* and antibiotic use in the hospital setting.

## Methods

### Ethics approval

The study obtained the necessary ethics approval from the Institutional Review Board Committee, HMC (protocol number IRGC-01-51-033), which complies with international ethical standards and regulations.

### Study design

The present study was conducted in Hamad General Hospital (HGH), which has a capacity of 603 beds, dealing with acute care, including intensive care beds, and which serves most of the inhabitants of Doha, Qatar. We evaluated the effect of ASP implementation in August 2015 on resistance patterns of *P. aeruginosa* over a 3 year period, between October 2014 and September 2017, compared with antibiotic consumption before and after programme implementation. The periods October 2014–September 2015 and October 2015–September 2017 were defined as pre-ASP and ASP analysis periods, respectively. Furthermore, we reviewed the trend of antimicrobial consumption across HGH wards since 2010.

### ASP

A formal ASP was introduced in August 2015 by a team consisting of an infectious diseases (ID) physician and a full-time pharmacist. The main strategy adopted by the ASP was a prospective-audit-and-feedback approach focused on IDSA recommendations for selected antimicrobials, based on their spectrum of activity and cost.^10^ Nominated restricted broad-spectrum antibiotics, including β-lactam/β-lactamase inhibitor combinations, third- and fourth-generation cephalosporins, glycopeptides, tigecycline, all carbapenems and all fluoroquinolones, were monitored and identified on a regular basis for the intervention teams to optimize prescribing. ‘Restricted-use’ antibiotics are defined in the HMC hospital’s antibiotic policy as those with a broad antimicrobial spectrum or high cost and which therefore need to be prescribed with caution. These indications are reviewed annually. The programmes are active 24 h a day, 7 days a week. All the ID physicians, including clinical fellows, have dedicated themselves to enforcing these programmes.

The patients treated with restricted-use antibiotics were identified by the pharmacy department. A daily record was kept of the active prescriptions of restricted-use antibiotics and new ones were communicated by email to the ASP/ID group. The ASP team attempted to: (i) identify ineffective or excessive antimicrobial coverage; (ii) ensure that the orders adhered to policies and guidelines; (iii) discontinue unnecessary double coverage; (iv) identify patients whose treatment could be converted safely from parenteral to oral therapy; and (v) suggest ID consultations for difficult and complex cases. Antibiotics were considered to be inappropriately prescribed if one or more of the following criteria were met: (i) a narrower-spectrum antibiotic could be used, based on the culture results; (ii) there was no infection present (i.e. bacterial colonization or an alternative explanation for the fever present); (iii) hospital antibiotic guidelines were not followed without valid reasons; and (iv) dosage, duration of therapy and/or empirical treatment choice was suboptimal according to the available guidelines.

### Antibiotic consumption

**Figure 1.**
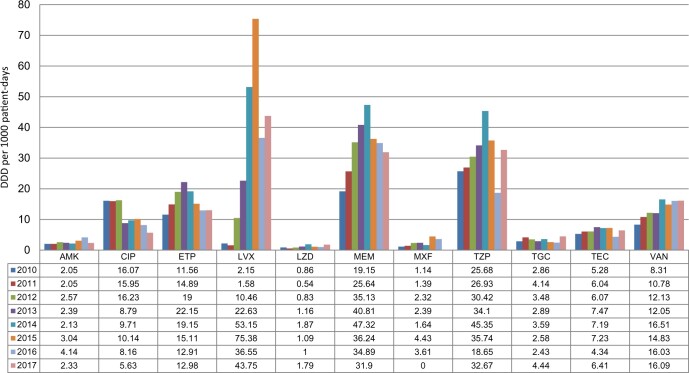
The evaluation of antibiotic consumption from HGH, between 2010 and 2017. AMK, amikacin; CIP, ciprofloxacin; ETP, ertapenem; LVX, levofloxacin; LZD, linezolid; MEM, meropenem; MXF, moxifloxacin; TZP, piperacillin/tazobactam; TGC, tigecycline; TEC, teicoplanin; VAN, vancomycin.

Utilization data for antimicrobials were obtained for 2010 to 2017 from a previously created database. Medication orders were summed per medication per month in grams using the total number of doses and dose per administration. The grams were then converted into census-normalized DDD per 1000 patient-days (DDD/1000 PD) using the WHO anatomical therapeutic chemical classification system of 2012.^15^ Bed-days were obtained from the hospital records. Totals for all ASP-focused and all systemic antibiotics were calculated per month. Changes in utilization were evaluated for the years before the ASP implementation and during the implementation period. The antimicrobial agents with antipseudomonal activities examined in this study included extended-spectrum cephalosporins (ceftazidime), piperacillin/tazobactam, carbapenems (meropenem), colistin, amikacin and antipseudomonal fluoroquinolones (ciprofloxacin and levofloxacin). Furthermore, we studied other antimicrobial agents such as aztreonam, daptomycin, ertapenem and linezolid (Figure 1).

We shared the antibiotic consumption data with prescribing units on a monthly basis.

### Clinical isolate identification and antimicrobial susceptibility testing

During a 3 year period, from October 2014 and September 2017, a total of 525 MDR *P. aeruginosa* strains were isolated. The bacterial identiﬁcation and initial antimicrobial susceptibility testing was done using the BD Phoenix automated system, while Lioﬁlchem MIC Test Strips (Lioﬁlchem, Rosetodegli Abruzzi, Italy) and the standard reference strains *Escherichia coli* ATCC 25922, *E. coli* ATCC 35218 and *P. aeruginosa* ATCC 27853 were used for quality control, as per recommendations of the CLSI, and the results were interpreted using the CLSI reference breakpoints. Testing was performed for eight antimicrobial agents: gentamicin, tobramycin, amikacin, cefepime, ciprofloxacin, piperacillin/tazobactam, meropenem and colistin. MDR *P. aeruginosa* isolates were deﬁned as having *in vitro* non-susceptibility to at least one agent from ≥3 antimicrobial classes.^16^ When necessary, the *P. aeruginosa* species identification was confirmed by MALDI-TOF MS using a Bruker Daltonics MALDI Biotyper (Billerica, MA, USA), according to the manufacturer’s instructions. A bacterial isolate of the same species and same antimicrobial susceptibility pattern in a patient isolated within 30 days, regardless of the site of isolation, was excluded. Isolates with major differences in susceptibility patterns were considered as new even if isolated within 30 days.

### Statistical analysis

All statistical analyses were done using Statistical Packages for Social Sciences v. 21.0 (SPSS Inc., Chicago, IL, USA). For categorical variables, data were expressed as number and percentage and were analysed by χ^2^ test or Fisher’s exact test, as appropriate. A *P* value of 0.05 or less was considered statistically significant.

## Results

Since 2010, we were able to obtain DDDs per 1000 PD for the studied antimicrobial agents, as illustrated in Figure 1. Overall, there was a significant increase in consumption from 2010 through to 2014 for most of the studied antibiotics. The largest total antibiotic consumption was seen for levofloxacin, followed by meropenem and piperacillin/tazobactam. We noted an increase in meropenem consumption to 247.1%, from 19.15 (2010) to 47.32 DDD/1000 PD (2014). Piperacillin/tazobactam use increased to 176.6%, from 25.68 (2010) to 45.35 DDD/1000 PD (2014). Furthermore, levofloxacin usage dramatically increased to 3506% from 2.15 (2010) to 75.38 DDD/1000 PD (2015) (Figure 1).

After the implementation of a restriction policy in 2015, the reduction in restricted antibiotics, in particular meropenem by 32.6%, is one of the highest reported in the literature. The total consumption of studied antibiotics was signiﬁcantly reduced by 23.9%. Antimicrobial consumption significantly decreased for piperacillin/tazobactam to 32.67 (2017) from 45.35 DDD (2014) (*P < *0.001), meropenem to 31.9 (2017) from 47.32 DDD (2014) (*P = *0.012) and ciprofloxacin to 5.63 (2017) from 9.71 DDD (2014) (*P *=* *0.015) (Figure 1).

**Figure 2.**
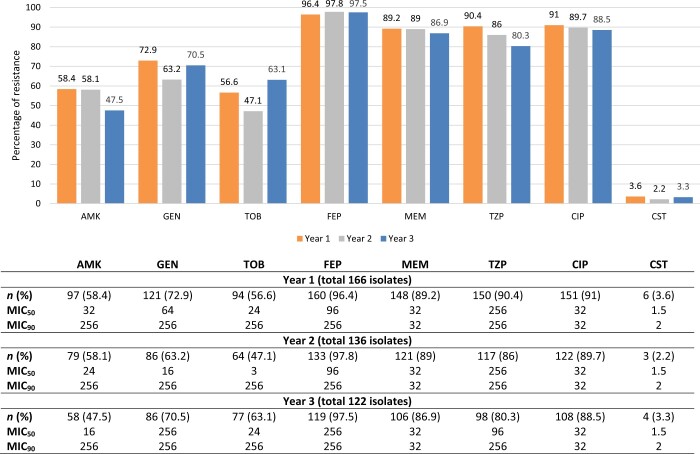
The resistance pattern of MDR *P. aeruginosa* between October 2014 and September 2017 in HGH. AMK, amikacin; CIP, ciprofloxacin; CST, colistin; FEP, cefepime; GEN, gentamicin; MEM, meropenem; TZP, piperacillin/tazobactam; TOB, tobramycin.

The study included 6501 consecutive non-duplicate isolates of *P. aeruginosa* (0.6% from a total of 1 054 672 microbiology samples) isolated from October 2014 to September 2017, from different clinical specimens taken from patients hospitalized in HGH (Table 1). The overall prevalence of *P. aeruginosa* isolates did not change signiﬁcantly over the study time (0.61% in 2015, 1844/300 577; compared with 0.55% in 2017, 2234/405 270) (Table 1). During our study period, we had 424 MDR *P. aeruginosa* isolated from different clinical specimens, as shown in Table 2. A high percentage of isolates were from tracheal aspirates (42.5%), skin and soft tissues (26.7%) and urine (24.8%) (Table 2). The prevalence of MDR *P. aeruginosa* signiﬁcantly decreased from 9% (166/1844) in 2015 (pre-ASP implementation) to 5.46% (122/2234) in 2017 (*P *=* *0.019). This was similarly linked to an observed fall in resistance patterns of MDR *P. aeruginosa* to piperacillin/tazobactam (90.4% to 80.3%), meropenem (89.2% to 86.9%), ciprofloxacin (91% to 88.5%) and amikacin (58.4% to 47.5%) (Figure 2). It must also be pointed out that consumption of anti-Gram-positive agents (both teicoplanin and vancomycin) consistently increased pre- and post-ASP implementation (Figure 2).

**Table 1.** The prevalence of MDR *P. aeruginosa* in HGH between 2014 and 2017

Period	Total no. of specimens^a^	Total no. of PA (%)	Total no. of MDR PA	Prevalence of MDR PA (%)	*P* value
Oct 2014–Sept 2015	300 577	1844 (0.61)	166	9	0.019
Oct 2015–Sept 2016	348 825	2423 (0.70)	136	5.60	
Oct 2016–Sept 2017	402 527	2234 (0.55)	122	5.46	
Total	1 054 672	6501 (0.62)	424	6.50	

PA, *P. aeruginosa.*

a Including all the negative culture specimens.

**Table 2.** Distribution and prevalence of MDR *P. aeruginosa* from HGH between October 2014 and September 2017

Infection site	Number of MDR *P. aeruginosa* (%)			
	Year 1 (Oct 2014–Sept 2015)	Year 2 (Oct 2015–Sept 2016)	Year 3 (Oct 2016–Sept 2017)	total
Respiratory	73 (44)	62 (45.6)	45 (36.9)	180 (42.5)
Skin and soft tissue^a^	42 (25.3)	33 (24.3)	38 (31.1)	113 (26.7)
Urine	41 (24.7)	32 (23.5)	32 (26.2)	105 (24.8)
Blood	4 (2.4)	4 (2.9)	4 (3.3)	12 (2.8)
Others^b^	6 (3.6)	5 (3.7)	3 (2.5)	14 (3.3)
Total MDR *P. aeruginosa*	166 (100)	136 (100)	122 (100)	424 (100)
MDR *P. aeruginosa* mucoid strains	14 (8.4)	18 (13)	9 (7.4)	41 (9.7)

a Skin and soft tissue: abscess, biopsy, ear, swab, tissue and wound.

b Other: eye, sterile body fluid, non-sterile body fluid, catheter tip.

## Discussion

Several strategies have been proposed to reduce and control antimicrobial use (formulary replacement or restriction, introduction of order forms, education of healthcare providers, prospective audit with intervention and feedback, and approval from an ID physician for antimicrobial drug prescription), but it has been suggested that antimicrobial restriction, either through formulary limitations or by the requirement for pre-authorization and justiﬁcation, is the most effective.^17^ This paper describes an ASP conducted over a period of 3 years at a secondary hospital. Our study showed the efficacy of an ASP on reducing the use of the targeted antibiotics and suppressing the emergence of resistant bacteria after we adopted a 48 h automatic stop for restricted antibiotics and, as a result, their non-dispensing by the pharmacy if not approved by an ID physician or the stewardship team. The design of this practice-based study was not to speciﬁcally measure the impact of any individual initiative with changes in resistance patterns, but rather to look at the impact of a multimodal stewardship programme on the prevalence of MDR *P. aeruginosa* and its susceptibility. Many published studies on antibiotic use and resistance have reported increased resistance with increased use of antibiotics.^18^ Our study is one of the few studies that show a decrease in meropenem, piperacillin/tazobactam and ciproﬂoxacin resistance among *P. aeruginosa* isolates with decreased meropenem, piperacillin/tazobactam and ciproﬂoxacin use. Slain and colleagues^19^ also reported temporal relationships between restricting antibiotic use and ICU resistance patterns. After the application of the programme for 24 months we saw a reduction in restricted antibiotics; in particular, meropenem use (which was the main target of the programme) decreased by 32.6%. The total consumption of studied antibiotics was signiﬁcantly reduced by 23.9%. The restriction policy that was applied mainly targeted the consumption of restricted antibiotics; the use of non-restricted antibiotics was affected only by the educational activities during daily visits and discussions with the teams.

We also investigated the prevalence and resistance to certain antibiotics in MDR *P. aeruginosa* isolates. In our study, 6501 *P. aeruginosa* clinical isolates were collected between October 2014 and September 2017. The ASP was strictly implemented in 2015 and the prevalence rate of MDR *P. aeruginosa* decreased from 9% to 5.46%, which was statistically significant. The study findings indicated that the trends in the resistance of *P. aeruginosa* to antimicrobial agents were influenced by the ASP implemented at HGH and that antibiotic usage was significantly related to the prevalence rate of MDR *P. aeruginosa*. Our data agreed with a study that looked into the relationship between carbapenem restriction in 22 university teaching hospitals and incidence rates of carbapenem-resistant *P. aeruginosa.*^20^ Even in the closed environment of a Saudi Arabian ICU, a recent carbapenem restriction policy programme proved to be useful in reducing consumption and in increasing susceptibilities of *P. aeruginosa* to imipenem and meropenem.^21^

Of note, there was a sustained consumption of anti-Gram-positive bacteria antibiotics for MRSA (vancomycin, linezolid and tigecycline), which probably did not contribute towards the declining rates of MDR *P. aeruginosa*. Although we studied the effect of the ASP on the susceptibility of MDR *P. aeruginosa*, showing favourable outcomes, we haven’t evaluated the effect on other GNB, particularly Enterobacteriaceae, which are associated with significant HAIs. Nevertheless, most of the broad-spectrum antibiotics used to treat *P. aeruginosa* are also used to treat HAI with Enterobacteriaceae. The expectation is that the resistance profile of other GNB will remain the same, as observed in other studies.^22^

Amongst the notable observations is the fall of piperacillin/tazobactam consumption during 2015–16, which eventually returned to baseline, most likely reflecting the temporary international shortage associated with the stream of production.^23^ Nevertheless, the consumption remained well below the level observed before introduction of the ASP in 2015.

It is also worth noting that one of the limitations of observational studies is the inability to establish causality. It has been previously established that propagation of AMR is not only linked to increased antibiotic consumption but also to poor infection control and prevention (ICP) measures.^24^ Conversely, good ICP measures might also lead to a reduction of AMR and improved susceptibility in the same manner as ASPs, particularly since the two processes are intimately linked.^24^^,^^25^ In our study, there were no major changes in the ICP programme timed with the intervention, which points towards the ASP as the main reason behind the observed results. Further limitations in this study include that there was no control for doses missed or not taken by the patients. Furthermore, we did not assess other risk factors for AMR, duration of hospital admission, readmission rate or causes of hospitalization. Finally, despite these limitations, we were still able to highlight a few trends by the temporal relationships of the antibiotic usage patterns and the resistance patterns.

### Conclusions

We demonstrated significant sustained reductions in both antimicrobial consumption, particularly broad-spectrum antibiotics, and the rates of MDR *P. aeruginosa* infections coupled with improved antibiotic susceptibility to standard treatment options. Epidemiological surveillance systems are necessary to monitor AMR organisms in conjunction with effective ASPs to deliver objectives. Such interventions are crucial for quality improvement and safeguarding patient outcomes.

## Supplementary Material

dlaa050_Supplementary_Data
