# Supplementary material for: Impact of an antimicrobial stewardship programme on antimicrobial utilization and the prevalence of MDR Pseudomonas aeruginosa in an acute care hospital in Qatar
Source: JAC Antimicrob Resist. 2020 Aug 7;2(3):dlaa050. doi: 10.1093/jacamr/dlaa050 (PMC8210253; doi:10.1093/jacamr/dlaa050)
Supplement: dlaa050_Supplementary_Data [file dlaa050_supplementary_data.zip › Reviewer report 1.docx]

**Reviewer report 1 (R0 – review of original submission)**

**PLEASE NOTE: JAC-AMR aims to publish your anonymous peer review comments with the final article for transparency. Please indicate in the box below whether you give your consent for us to publish your peer review comments for this article.**

Yes

**Synopsis**

**1. If the article contains original data, does the synopsis adequately reflect the manuscript as a whole, with the most important aspects described (of the introduction, methods and key results, and a balanced conclusion)? If NO, what is required to improve the synopsis? Please use the box below.**

No

An abstract should be complete in itself and should clearly summarise the findings of the manuscript. The objective in the abstract does not mention the effect of Antimicrobial stewardship (ASP) on prevalence of MDR PA. It rather mentions the effect on trends of antimicrobial resistance in P.aeruginosa.
The methodology described in the abstract is not clear and is incomplete in description. Trends of antimicrobial resistance in P.aeruginosa have been compared to measurements of antimicrobial consumption. However no correlation between impact of ASP and prevalence of P.aeruginosa in the before and after ASP introduction has been shown.
The results in the abstract need to be rewritten to highlight the findings of the study. The results also do not correlate with that of the text and thus clarity is not evident.

**Introduction**

**2. Does the introduction provide the necessary background information for readers to understand the article? (e.g. does it describe the problem, current state of knowledge, etc.) If NO, what is required to improve it? Please use the box below**

No

**3. Is the research hypothesis and/or aims/objectives of the study clearly stated in the introduction? If NO, what is required to improve it? Please use the box below**

No

**4. Is there superfluous/unnecessary text in the introduction? (Only text that is required for readers to understand why the study has been done should be included). If YES, what text is superfluous and should be removed? Please use the box below**

Yes

Considering the answers above and any other comments/advice you may have, please indicate what is required to improve the Introduction:

Although the topic has been introduced to highlight the problem of rising antimicrobial resistance (AMR), however the introduction of the efficacy of different ASP programmes in reducing AMR has not been defined and introduced well. The gap analysis in performing the current study is not mentioned. It is also not clear why P.aeruginosa was particularly chosen as the study bacteria.

Line 71  it is not clear of the bacteria to which AMR for Carbapenems has increased.
 Superfluous data (Line 67-69);
Overall , the introduction has not been organised well and thoughts jump from here and there.

**Methods**

**5. Were the methods used suitably robust and appropriate for the research question(s) and/or the aims/objectives of the study? If NO, the article should be rejected, but please advise the authors accordingly in the free-text box below and complete the review in full**

No

**6. Are the methods described in enough detail so the reader can understand what was done and interpret the results and conclusions appropriately?**

No

**7. Are the methods described in language that a reader who does not specialize in the area of the study/methods used will understand? For example, are the statistical approaches used described in language that non-statistician healthcare professionals will understand? If NO, please indicate what is required to improve the language in the methods section in the box below**

Yes

Considering the above and any other comments/advice you may have, please indicate other aspects of the methods section that could be improved in the free-text box below)

1. Although 3 years has been taken the study time period however time period of pre intervention and ASP is not mentioned clearly
2. MDR-PA has not been described and defined
3. List of antibiotics monitored for defining MDR PA has not been mentioned
4. List of restricted antibiotics which were  included for monitoring have not been mentioned
5. Type of ASP intervention undertaken is not mentioned in the methods
6. Clarity on microbiological identification and susceptibility testing methods is not there. A detailed description must be provided as to the methods.
7. It is not clear about the antimicrobial usage records (Purchase or Consumption records) .Reference for the methodology used for calculation of of antibiotic consumption in DDD/1000 patient days is not evident in references.
8. Line 103 mentions confirmation by MALDITOF. but it is not clear what is being confirmed (Identification or sensitivity)
9. Overall many grammatical mistakes have been identified in the statistical analysis section (Line 110 and line 114)

**Results**

**8. Given the methods used, are results described accurately, in enough detail and in an appropriate format to adequately inform the discussion section and the authors/readers conclusions? If NO, please indicate what is required to improve the results section in the box below**

No

**9. Based on what is described in the methods section, are data/results (that are likely to be available to the authors) missing from the current draft that you think should be presented to improve the manuscript?**

Yes

**10. Are Tables/Figures clear, required and do they add/complement the results text (they should not simply repeat what is in the text or vice versa)? If NO, please indicate what is required to improve the Tables/Figures in the box below**

No

Considering the above and any other comments/advice you may have, please indicate what is required to improve the results section:

1. Trends of antibiotic consumption from 2010 have been described in results section. However no mention of the same has been written in the material and methods section or introduction.
2. The antibiotics monitored for AMR have not been described clearly in results section.
3. The results of the low end antibiotics and high end antibiotics have not been related.
4. The abbreviations mentioned in the figure 2 do not correlate with the full forms  mentioned below for FEP, MEM, TZP.
5. Wherein the antibiotic consumption measurement has been in DDD/1000 patient days, Figure 1 mentions as DDD/1000 Bed day. The discrepancy need revision.

**Discussion**

**11. Are the discussion and any conclusion(s) reasonable given the stated hypothesis/aims/objectives and results? If NO, please indicate how to improve the discussion in the box below**

No

**12. Is there superfluous/unnecessary text in the discussion? (Only text that directly relates to the results and understanding thereof in the context of existing published literature and the limitations of the study should be included.) If YES, please indicate what should be removed to improve the discussion in the box below**

Yes

**13. Is the Limitations sub-section adequate and reflective of the actual limitations of the study? ? If NO, please indicate how to improve the Limitations section in the box below**

Yes

Considering the above and any other comments/advice you may have, please indicate how to improve the discussion section:

Superfluous text: Line 134- 141
Lines 142- 144: It is not clear how many and which prescriptions were refused restricted antibiotics after 48 hours, and what was given as an alternate.
Lines 145 - 147: It is not clear from where these figures have come. No relation in the results was identified.
Line 155: HGH must have the full forms
Line 156: The study aims on finding the prevalence in the beginning, but this line mentions the incidence rate.
Line 185: The sustainability time period has not been mentioned and the method not specified to monitor in the methods section.

**General**

**14. Is referencing throughout the manuscript satisfactory? (for instance are there statements for which you believe a supporting reference should be cited?) If NO, please indicate how to improve referencing in the box below**

No

**15. Is the language used (i.e. spelling, grammar, etc.) of high enough quality to be published as is? If NO, please indicate how to improve the language within the article in the box below**

No

**16. Are ethics/other approvals appropriate/adequate and satisfactorily described? ? If NO, please indicate how to improve this in the box below**

Yes

Considering the above and any other comments/advice you may have, please advise the authors how to improve these aspects of the article.

Line 96: Methodology for calculating antibiotic consumption based on WHO (DDD/100 patient days ) needs to be referenced.
There are many grammatical mistakes in the overall manuscript. They must be revised

**Opinion and review articles only**

**17. Please advise the authors in the box below on how to improve their opinion or review article:**

1. Please revisit the title, aims and objectives, and methodology to have a link in all the three areas.
2. The results must be clearly in line with the methodology, For eg. prioritizing results which were the main aim of the study. Ancillary results and additional findings must follow and the least important results must be written at the last.
3. All results narrated in results section only, must be discussed without adding any additional result in discussion section.

|  **Recommendation** | |
| --- | --- |
|  | Accept (as is with no revisions) |
|  | Minor revision (with anticipation of subsequent acceptance if revisions are adequate) |
|  | Major Revision |
|  | Reject |

| **Would you be willing to review a revision of this manuscript?** | |
| --- | --- |
|  | Yes |
|  | No |

**Comments**

 Comments to the Author

If you have any additional comments to the authors about any aspect of the manuscript not covered in the structured review above, please place these in the box below. If you have no additional comments, please say “No additional comments” in box.

No additional comments
